# Cognitive dysfunction in chronic Chagas disease cardiomyopathy

**DOI:** 10.1590/S1980-57642009DN30100006

**Published:** 2009

**Authors:** Jesângeli Sousa Dias, Amanda M. Lacerda, Rodrigo M. Vieira-de-Melo, Leila C. Viana, Pedro A.P. Jesus, Francisco J.F.B. Reis, Ricardo Nitrini, Helenice Charchat-Fichman, Antônio A. Lopes, Jamary Oliveira-Filho

**Affiliations:** 1 Cardiomyopathy and Stroke Clinics, Federal University of Bahia, Salvador, BA, Brazil.; 2 Behavioral and Cognitive Neurology Unit, University of Sao Paulo, São Paulo, SP, Brazil.

**Keywords:** Chagas disease, American trypanosomiasis, cognition, cerebrovascular disorders

## Abstract

Chagas disease (CD) remains a major cause of stroke in developing countries, but cognitive repercussion of CD has not been well studied.

**Objective:**

To compare the frequency and pattern of cognitive dysfunction in patients with CD cardiomyopathy (CDC) and other cardiomyopathies (OC).

**Methods:**

We studied 37 patients with CDC and 42 patients with OC with similar age, educational level and cardiac systolic function. Cognitive tests were applied to both groups by a single examiner blinded to CD status. Logistic regression multivariable models were constructed to ascertain predictors of cognitive dysfunction for each test.

**Results:**

Cognitive dysfunction was detected in 9 (24%) CDC patients and 6 (14%) OC patients by Mini Mental State Exam (MMSE) corrected for educational level. Independent predictors of abnormal MMSE (p<0.05) included stroke history (OR=5.51; 95% CI=1.27–24.01) and digoxin use (OR=0.23, 95% CI=0.06–0.89), while CD showed a trend toward statistical significance (OR=4.63; 95% CI=0.87–24.73, *p=*0.07). Delayed recall of Rey’s Complex Figure Test was significantly worse in CD patients, where this remained a significant predictor in the multivariable analysis (OR=4.67; 95% CI=1.23–17.68).

**Conclusions:**

Cognitive dysfunction is frequent in Chagas disease and should be considered as an outcome measure in Chagas disease studies.

## Abstract (pt)

A doença de Chagas (DC) permanence uma causa importante de acidente vascular cerebral em países em desenvolvimento, mas a repercussão cognitiva dessa doença não tem sido bem estudada.

**Objetivo:**

Comparar a frequência e padrão de disfunção cognitiva em pacientes com cardiomiopatia associada à DC (CDC) em comparação com outras cardiomiopatias (OC).

**Métodos:**

Foram estudados 37 pacientes com CDC e 42 portadores de OC com idade, nível de instrução e função sistólica cardíaca semelhantes. Testes cognitivos foram realizados em ambos os grupos por um único investigador cegado quanto ao diagnóstico de DC. Modelos de regressão logística multi-variável foram construídos para detectar preditores de disfunção cognitiva para cada teste.

**Resultados:**

Disfunção cognitiva foi detectada em 9 (24%) pacientes com CDC e 6 (14%) com OC pelo Mini Exame do Estado Mental (MEEM) corrigido pelo nível de instrução. Preditores independentes de MEEM anormal (p<0,05) foram: história de AVC (OR=5,51; IC 95%=1,27–24,01) e uso de digoxina (OR=0,23, IC 95%=0,06–0,89); DC mostrou tendência a significância estatística (OR=4,63; IC 95%=0,87–24,73, *p=*0,07). Pacientes com CDC apresentaram pior desempenho na evocação tardia da figura complexa de Rey. Neste teste, a DC se manteve um preditor significante de disfunção cognitiva na análise multivariável (OR=4,67; IC 95%=1,23–17,68).

**Conclusões:**

Disfunção cognitiva é frequente na doença de Chagas e deve ser considerada como desfecho quantificado nos estudos da doença de Chagas.

Chagas disease (CD) is a chronic infection caused by the protozoal parasite *Trypanosoma cruzi*, affecting primarily the heart and/or the digestive system.^1^ Over 16 million people are infected worldwide,^2^ mostly in South America, but cases have also been described in immigrants,^3^ raising concerns over possible blood-borne transmission.^4^

A cerebral form of CD was suggested in Chagas’ original series,^5^ but later refuted by other authors, who rarely identified active brain inflammation, attributing most brain involvement to cardioembolic stroke.^6-8^ However, cases of active brain inflammation have been described in immune suppressed patients,^6^ and brain atrophy was found more frequently in CD patients compared to patients with idiopathic dilated cardiomyopathy in one pathological series.^8^ The clinical repercussion of these pathological findings have been seldom explored – only one study has investigated cognition in CD but did not correct for the presence of congestive heart failure,^9^ a variable known to affect cognition.^10-12^ In the present study, our objectives were:

1) to investigate the frequency and pattern of cognitive abnormalities in patients with CD cardiomyopathy (CDC) as compared to other cardiomyopathies (OC); and
2) to determine the predictors of cognitive dysfunction in both groups.

## Methods

A random sample of patients was evaluated from a cardiomyopathy clinic in a university-based outpatient center. Approximately 50 patients are seen on a weekly basis by this clinic. Patients who arrived in the clinic each day were screened through chart reviews for inclusion and exclusion criteria. Potential candidates were then consented for study participation. In cases of illiteracy, a caregiver was asked to provide signed consent after a full verbal explanation of the study to both patient and caregiver. Echocardiographic inclusion criteria included an ejection fraction <40% or a borderline-low ejection fraction (40–49%) in the presence of dilated heart chambers (left ventricle systolic diameter >45 mm and diastolic diameter >55 mm). Echocardiograms had to have been performed within one year of study entry. Exclusion criteria included physical inability to read or write (e.g., blindness), renal failure (defined by serum creatinine above 1.5 mg/dl), history of liver failure, previous cardiac arrest, history of chronic obstructive pulmonary disease and untreated hypothyroidism (defined by serum thyrotropin above 5 mU/L). Previous stroke history was not an exclusion criterion provided current general neurological exam was normal. All patients were subjected to a structured interview with prospective collection of demographic data, cerebrovascular risk factors, medications currently used, most recent electrocardiogram and transthoracic echocardiogram. New York Heart Association heart failure functional class, supine blood pressure and heart rate were obtained on admission. Cardiomyopathy etiology was defined by the attending cardiologist and Chagas disease confirmed by appropriate serology (hemaglutination or immune-fluorescence tests). A full neurological examination including the NIH Stroke Scale was performed by an investigator certified in applying the scale. The present study was approved by the hospital ethics committee.

A structured cognitive evaluation, lasting approximately 90 minutes, was performed by a single investigator who was not involved in patient screening and who remained blinded to CD-status. Cognitive tests included the Mini Mental State Examination (MMSE),^13^ Nitrini’s Brief Cognitive Screening Battery (BCSB) (which includes visual perception, incidental memory, delayed memory – after 5 minutes, drawing of a clock and verbal fluency – animal list),^14-15^ digit and visual span (forward and backward),^16^ Rey’s Auditory Verbal Learning Test,^17^ Rey-Osterreith Complex Figure Test (ROCFT),^18^ computerized tests to measure mental processing speed, and the Hospital Anxiety and Depression Scale.^19^ The ROCFT was scored based on previously published criteria, where drawing strategies are categorized into seven patterns, four of which are classified as “normal” and the remaining as “abnormal”.^18^ Cut-off values for abnormal MMSE scores corrected for educational level were used based on previous work validating the test in the Brazilian population: 18 for illiterates, 23 for those with 1–7 years of schooling and 26 for those with 8 or more years.^20,21^

For statistical analysis, Student’s t test or the Mann Whitney U test were used as appropriate for continuous variables, and Fisher’s exact test for categorical variables was employed to compare groups with CD and OC, as well as patients with or without abnormal MMSE. Any variable with a P-value <0.1in univariable analyses was included in multivariable logistic regression models to predict abnormal MMSE, as was any cognitive test which yielded a significant difference between CD and OC.

## Results

The study screened 92 patients, 13 of whom were excluded for the following reasons: two refused to complete the cognitive tests, two were later found to have renal failure, one had a previous history of cardiac arrest, and eight had echocardiograms older than one year that, when repeated, did not fulfill inclusion criteria. The final sample was therefore composed of 79 patients – 37 with CD and 42 with OC, evaluated between May, 2004 and June, 2005. Cardiomyopathy etiologies in the OC group were hypertensive dilated cardiomyopathy in 18 (42%), dilated ischemic cardiomyopathy in 10 (23%), idiopathic dilated cardiomyopathy in 10 (23%) and other dilated cardiomyopathies in 4 (9%).

Univariable analyses comparing CD and OC groups are presented in Table 1. Patients with OC more often had a previous diagnosis of hypertension and coronary artery disease, and had a higher blood pressure and heart rate on admission. Antiplatelet medications and statin use were more frequent among OC patients whereas amiodarone use was more frequent among CD patients. No patient was receiving sedative medication at the time of cognitive testing. Univariable analyses comparing patients with normal or abnormal MMSE corrected for educational level identified the following variables as associated with abnormal scores: previous stroke history (*p=*0.086), age (*p=*0.075) and lack of digoxin use (*p=*0.066).

**Table 1:** Clinical and demographic characteristics of 79 patients with cardiomyopathy.

	Chagas disease cardiomyopathy (n=37)	Other cardiomyopathies (n=42)	*p*
Age, years (mean±SD)	52.5 (±9.7)	56.3 (±16.1)	0.16
Male, n (%)	23 (62.2)	23 (54.8)	0.64
Years of education, median (range)	4 (0-11)	4 (0-11)	0.29
NYHA heart failure functional class, n (%)			0.47
I	15 (41.7)	21 (50.0)	
II	15 (41.7)	16 (35.7)	
III	6 (16.7)	6 (14.3)	
Ejection fraction, %	37.4 (±8.7)	36.4 (±6.9)	0.47
Anxiety*, n (%)	15 (40.5)	15 (36.6)	0.72
Depression*, n (%)	14 (37.8)	9 (22)	0.12
Hypertension, n (%)	12 (32.4)	28 (66.7)	0.002
Diabetes, n (%)	3 (8.1)	5 (11.9)	0.71
Coronary artery disease, n (%)	5 (13.5)	17 (40.5)	0.008
Atrial fibrillation, n (%)	5 (13.5)	1(2.4)	0.09
Smoking, n (%)	2 (5.4)	2 (4.8)	1.0
Ischemic stroke, n (%)	6 (16.2)	6 (14.3)	0.81
Hypercholesterolemia, n (%)	1 (2.7)	7 (16.7)	0.06
Daily alcohol use, n (%)	2 (5.4)	1(2.4)	0.59
Digoxin use, n (%)	24 (75)	27 (68)	0.49
Systolic blood pressure, mmHg	116.3 (±22.6)	129.3 (±22.1)	0.01
Diastolic blood pressure, mmHg	79.9 (±15.3)	81.1 (±12.3)	0.57
Heart rate, bpm	64.9 (±11.5)	70.6 (±11.5)	0.03

* Hospital Anxiety and Depression Scale19; NYHA, New York Heart Association.

Cognitive test results are presented in Table 2. Abnormal MMSE score was found in 9 (24%) CD patients and 6 (14%) OC patients (*p=*0.2). For the remaining tests, no significant differences were detected between the two groups, except for ROCFT. Patients with CD obtained lower scores and more frequently used abnormal strategies on delayed recall of ROCFT compared to OC patients (68% vs. 37%, *p=*0.011).

**Table 2:** Cognitive test scores in patients with Chagas disease cardiomyopathy (CDC) and other cardiomyopathies (OC).

	CDC, median (range)	OC, median (range)	*p*
MMSE score	25 (16-30)	26 (18-30)	0.09
Digit span forward	4 (0-8)	5 (1-9)	0.90
Digit span backward	3 (0-7)	3 (0-7)	0.07
Visual span forward	7 (3-9)	7 (3-11)	0.70
Visual span backward	3 (1-10)	4 (0-11)	0.30
Clock drawing	5 (2-7)	5 (1-10)	0.40
Verbal fluency	16 (5-27)	16 (6-34)	0.70
Immediate memory BCSB	7.6 (5.6-9.6)	8.0 (6.3-9.6)	0.10
Delayed memory BCSB	9 (5-10)	9 (4-10)	0.10
Recall BCSB	10 (9-10)	10 (10)	0.10
Immediate memory RAVLT	41 (15-59)	40.5 (24-55)	0.30
Delayed memory RAVLT	7 (0-14)	8 (2-13)	0.30
Recall RAVLT	11 (0-15)	11 (1-15)	0.90
ROCFT copy	14 (0-25)	17.7 (0-34)	0.13
Delayed recall ROCFT	4 (0-16.5)	6 (0-22.5)	0.02
ROCFT copy time (seconds)	386 (126-945)	392 (110-1000)	0.85

BCSB, Nitrini's Brief Cognitive Screening Battery; RAVLT, Rey's Auditory Visual Learning Test; ROCFT, Rey-Osterreith Complex Figure Test.

Multivariable predictors of abnormal cognition defined by MMSE are shown in Table 3. The only variable with a significant association to abnormal cognition was a previous stroke history (OR=5.51; 95% CI=1.27–24.01), while digoxin use had a protective effect (OR=0.23; 95% CI=0.06–0.89). Chagas disease (*p=*0.07) and age (*p=*0.08) showed a statistical trend as predictors of abnormal cognition.

**Table 3:** Predictors of abnormal Mini Mental State Exam corrected for educational level. Odds ratios (OR) and 95% confidence intervals (CI) are expressed as non-adjusted and adjusted values (multivariable logistic regression).

Variable	OR (95% CI)	
	Non-adjusted	Adjusted
Coronary artery disease	1.38 (0.41-4.62)	1.16 (0.23-5.60)
Hypertension	2.26(0.70-7.38)	1.69 (0.37-7.02)
Chagas disease cardiomyopathy^†^	1.92 (0.61-6.06)	4.63 (0.87-24.73)
Digoxin use*	0.29 (0.09-0.93)	0.23 (0.06-0.89)
Stroke history*	6.44 (1.70-24.40)	5.51 (1.27-24.01)
Age^††^	1.04 (0.99-1.09)	1.06(0.99-1.38)

* *p*<0.05;

† *p*=0.07;

†† *p*=0.08

Since abnormal late recall of ROCFT was significantly different between CD and OC patients, we performed another multivariable analysis to correct for potential confounders of this test (Table 4). When correcting for age, educational level, digoxin use, and history of hypertension, stroke or coronary artery disease, CD remained a significant predictor of an abnormal test (OR=4.67; 95% CI=1.23–17.68).

**Table 4:** Predictors of abnormal delayed recall on Rey-Osterreith Complex Figure Test. Odds ratios (OR) and 95% confidence intervals (CI) are expressed as non-adjusted and adjusted values (multivariable logistic regression).

Variable	OR (95% CI)	
	Non-adjusted	Adjusted
Coronary artery disease	0.88 (0.31-2.46)	0.82 (0.23-2.94)
Hypertension	2.15 (0.85-5.46)	2.15 (0.44-10.48)
Chagas disease cardiomyopathy*	3 (1.16-7.74)	4.67 (1.23-17.68)
Digoxin use	0.57 (0.21-1.59)	0.39 (0.11-1.35)
Stroke history	2.59(0.71-9.52)	2.15 (0.44-10.48)
Educational level^†^	0.42 (0.23-0.73)	0.41 (0.20-0.84)
Age	1.02 (0.98-1.06)	1 (0.95-1.05)

* *p*=0.02;

† *p*=0.01.

## Discussion

Cognitive repercussion of Chagas disease has been seldom explored. We found only one published study, comparing patients with CD to a normal control group, in which various cognitive domains such as non-verbal reasoning, orientation, problem-solving and sequencing were abnormal.^9^ However, these abnormal results may have been due to heart, and not brain involvement, since congestive heart failure is known to affect cognition.^11-12,22^ Our study is the first to study cognition in CD, correcting for the presence of cardiomyopathy as a confounder. Thus, we attempted to clinically evaluate the direct effects of CD on the brain. Patients with CD are usually young and have few risk factors for atherosclerosis, making CD an interesting model to study the effects of inflammation and/or silent embolism on the brain.

In our patients with CD, abnormal cognition was found in one-quarter of patients. This is a substantial proportion, considering that over 80% of patients were in mild heart failure classes (I or II). When correcting for other potential predictors of abnormal cognition, CD showed a trend as an independent predictor (*p=*0.07). A larger sample will be necessary to answer this important question, since impaired cognition is related to poor patient compliance with medications and increased mortality from heart failure.^23,24^

Two other variables predicted abnormal cognition: previous stroke history and lack of digoxin use. Although only 12 patients with both previous stroke history and a normal neurological exam were included in our sample, our findings showed that detailed cognitive testing may still be abnormal in these patients. This is not surprising, since cerebrovascular disease is one of the main causes of dementia in adults. The protective effect of digoxin was unexpected and the reasons for this association are speculative. Cognitive abnormalities have been previously associated with increasing blood levels of digoxin,^25^ but this may have reflected worsening heart failure. Recently, endogenous hypothalamic digoxin has been postulated to be involved in normal cognition.^26,27^ Whether exogenously administered digoxin may improve cognition warrants future study.

When comparing patients with CD and OC, ROCFT performance on delayed recall was the only abnormality detected, yielding worse scores in patients with CD. Even after correcting for age, educational level, digoxin use, and cerebrovascular risk factors, CD remained an independent predictor of an abnormal ROCFT.

Two main reasons could explain this association. First, active or previously active brain inflammation could play a role in chronic forms of CD. Although active inflammation is rarely detected on brain autopsy,^6^ a greater proportion of brain atrophy has been detected in CD patients when compared to patients with idiopathic dilated cardiomyopathy, possibly indicating previously active inflammation.^8^ Brain atrophy or active inflammation could predispose CD patients to more frequent or severe cognitive deficits.

A second possibility to explain our findings could be silent ischemic stroke. Chagas disease is a highly embolic condition due to a high proportion of left ventricular thrombus, a hallmark of the disease.^28^ The association between CD and stroke is independent of cardiac disease severity.^29^ Recently, it has been suggested that silent ischemia may be more frequent in right-hemisphere stroke given that cognitive abnormalities such as anosognosia, hemi-neglect and visuo-spatial deficits are more difficult for patients and caregivers to recognize.^30^ The ROCFT is widely used to evaluate visual perceptual organization and visual memory. Thus, it is possible that in our population, composed mostly of patients without known history of stroke, silent ischemia to the right hemisphere may have been responsible for the abnormal findings in ROCFT.

Another significant predictor of cognitive test abnormality was educational level. This particular population was composed mainly of low educational level patients, with 60.5% of the sample having less than 4 years of formal training. This finding did not affect our conclusions regarding CD in comparison to OC patients, since educational level was similar in both groups and ROCFT scores were corrected for educational level on multivariable analysis. Educational level interferes with verbal, constructive and visual-spatial abilities.^31^ Illiteracy seems to interfere with brain development itself, since illiterate patients perform worse even on simple nonverbal tests.^15,32^

There are several limitations to our study. First, our population was restricted to patients with congestive heart failure, so our findings cannot be extrapolated to other forms of Chagas disease. Second, no formal sample size calculation was performed in this exploratory hypothesis-generating study, with the relatively small sample size increasing chances of a type II error. Multiple comparisons may have also increased the chances of a type I error. Third, the higher frequency of cerebrovascular risk factors such as hypertension, coronary artery disease and dyslipidemia in the OC patients may have negatively affected cognitive tests in this group, decreasing our ability to detect differences in both groups. Finally, no neuroimaging was performed in this study, which decreases our ability to identify the exact mechanisms underlying the cognitive abnormalities observed, including “silent” ischemia.

In conclusion, Chagas disease was associated with more frequent subtle abnormalities on cognitive testing in comparison to patients with other cardiomyopathies. Further investigation into the mechanisms responsible for this finding could impact our strategies toward primary prevention of cognitive changes in Chagas disease patients.

## Figures and Tables

**Figure 1:**
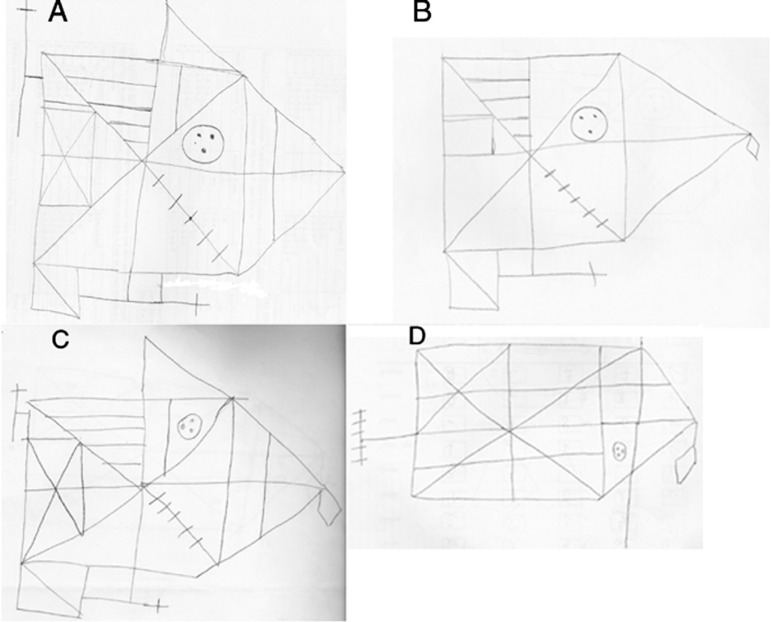
Example of Rey-Osterreith Complex Figure Test. A and B to refer to immediate copy and delayed recall (after 20 minutes), respectively, in a 25-year-old patient with idiopathic dilated cardiomyopathy with 10 years of formal education. Total scores were 32 and 22.5 for A and B, respectively. C and D refer to immediate copy and delayed recall, respectively, in a 41-year-old patient with Chagas disease cardiomyopathy and 9 years of formal education. Total scores were 25 and 11 for C and D, respectively. The patient with Chagas disease performed significantly worse on delayed recall (D) compared to the patient without Chagas disease (B), despite a similar educational level.
